# 

**Published:** 1883-04

**Authors:** 


### CONTENTS.

### COMMUNICATIONS.

On Some Scientific Problems of Dental Surgery........................ 151
A Simple Stomatoscope ........................................................ 184
Degeneration of the Teeth.................................................... 186
Historical Sketch of the Mad River Dental Society.................... 189

### PROCEEDINGS.

Thirty-ninth Annual Meeting of the Mississippi Valley Association of Dental Surgeons ........................................................ 162

### EDITORIAL.

Meeting of Dentists at Louisville ........................................ 194
University of Michigan........................................................ 195
Dental Department of the University of Maryland .................... 196
Change of Time.................................................................... 198
Ohio College of Dental Surgery............................................. 198
Merited Honor...................................................................... 200
There are Two...................................................................... 200
N. O. D. A............................................................................ 201
Removal .............................................................................. 201
Chicago Dental Society Election .......................................... 202
Mad River Dental Society..................................................... 202

